# Application of Information Theory to Computer Vision and Image Processing

**DOI:** 10.3390/e26020114

**Published:** 2024-01-26

**Authors:** Wendy Flores-Fuentes, Oleg Sergiyenko, Julio C. Rodríguez-Quiñonez, Jesús E. Miranda-Vega

**Affiliations:** 1 Facultad de Ingeniería, Universidad Autónoma de Baja California, Mexicali 21100, Mexico; julio.rodriguez81@uabc.edu.mx; 2 Instituto de Ingeniería, Universidad Autónoma de Baja California, Mexicali 21100, Mexico; srgnk@uabc.edu.mx; 3 Tecnológico Nacional de México, IT de Mexicali, Mexicali 21376, Mexico; elias.miranda@itmexicali.edu.mx

## 1. Introduction

Our perception of the world is the product of the human visual system’s complex optical and physical process. When we open our eyes, light stimuli enter our pupils, which are the gateway to our visual experience.

These incoming rays of light then pass through the various structures of the eye, such as the cornea and lens, which help the light to focus onto the retina. The retina, located at the back of the eye, is a crucial component in the process of perceiving the world. It is composed of specialized cells called photoreceptors, namely rods and cones. Rods are responsible for vision in low-light conditions and help us perceive shades of gray, while cones enable us to see colors and function best in bright light.

As light reaches the retina, the photoreceptors initiate a remarkable transformation. They convert the incoming light into electrochemical signals that can be transmitted to the brain through the optic nerve. This process involves the absorption of light by pigments in the photoreceptor cells, triggering a cascade of chemical reactions that generate electrical impulses.

The transmitted electrical signals, laden with visual information, travel along the optic nerve to the visual cortex in the brain. Here, the incoming data undergo a complex process that allows us to organize, interpret, and analyze the information received. The brain seamlessly integrates this visual input with other sensory cues, such as auditory and tactile information, to create a coherent and multi-dimensional perceived reality. It is important to note that perception is not a direct replication of the external world but rather a constructed representation based on the available sensory input. Factors like individual differences in perception, attention, and previous experiences can shape how we interpret and make sense of the visual information received.

The process underlying humans’ perception of the world involves intricate interplay between the eye’s optical components, the retina’s photoreceptors, and the brain’s complex neural networks. Together, they transform light into meaningful visual experiences, allowing us to navigate and interact with the world around us.

In a similar way to the intricate optical and physical processes of human vision, machine vision serves as the “eyes” of cybernetic systems. Machine vision refers to technology that enables machines to process and interpret visual information, much like how human eyes perceive and understand their surroundings, facilitating the coexistence of the virtual and real world in our daily lives. Cybernetic systems are involved in multiple disciplines, and they address the emerging challenges of managing the information provided from the virtual and physical world to offer solutions that adhere to human needs and demands [1]. Machine vision, as a part of cybernetic systems, is vital for enabling these systems to navigate and interact within both virtual and real-world environments in diverse applications, including in smart cities, factories, and homes, via monitoring, analyzing, and controlling machinery, devices, and objects based on end-to-end data collected by smart sensors connected to the internet and a cloud network [2].

Machine vision systems are based on technologies that strive for seamless integration into our lives, are driven by creativity and a global perspective, are enabled by the power of the intelligent interconnectivity of several surrounding environments related to an application [3], and are continuously evolving due to ongoing research and technological innovations, including improvements in efficiency, accuracy, and the development of novel information theories for computer vision and image processing models [4,5,6] and applications like those based on collaborative multi-agent approaches applied mainly in swarm robotics [7].

This remarkable collaboration between agents and the fusion of their information has been made possible through the advancement of sensor technologies and sophisticated systems that acquire and process vast amounts of information through the Internet of Things [8,9,10]. Machine vision relies on a harmonious amalgamation of optoelectronics devices, sensors, cameras, and technical vision systems. These components work together to capture visual data, which form the foundation for subsequent analysis and interpretation. In this era of big data, the main technological challenges are related to handling high-throughput tasks that are both complex and efficient, which requires the development of new materials, new operational principles, and new designs to fulfil the requirements. These developments require the mimicking of the relationship between the structures and functions found in the human visual system, demonstrating significant potential for efficiently processing optical information while consuming minimal power [11].

The field of machine vision encompasses a diverse range of technologies and methodologies, including artificial intelligence algorithms like deep learning algorithms and neural networks for recognizing [12] and classifying objects in images or videos [13], enhancing image quality and reducing noise in images [14], and 3D vision and depth sensing [15]. These algorithms are robust and adaptable, and they are used in embedded systems [16], robust control mechanisms [17], inertial navigation systems, robotics, interconnectivity, big data applications, and cloud computing applications [18]. These elements are at the core of machine vision advancements, enabling cyber–physical systems to collaborate with humans in both their real and virtual environments and activities [19].

Sensors play a pivotal role in machine vision, acting as the first point of contact for acquiring data from the environment. These carefully designed and calibrated sensors are capable of detecting and measuring various physical properties, such as light, temperature, pressure, and motion. The acquired data are then processed through sophisticated algorithms and computer vision techniques, which extract meaningful information and patterns from the raw sensory input [20].

Artificial intelligence (AI) algorithms, a driving force behind machine vision, allow systems to understand, interpret, and make decisions based on the captured data. These algorithms leverage deep learning, neural networks, and pattern recognition to discern objects, recognize faces, analyze scenes, and even predict future events. The integration of AI algorithms empowers machine vision systems to adapt and learn from their interactions with the environment, continuously improving their performance and enhancing their ability to assist humans in diverse tasks [21].

Embedded systems and robust control mechanisms ensure the seamless integration and synchronization of various components within machine vision systems. These systems coordinate the operation of sensors, cameras, actuators, and other peripherals, ensuring precise data acquisition and processing. By tightly controlling the system’s behavior, machine vision can deliver accurate and reliable results, even in challenging and dynamic environments.

Interconnectivity, big data, and cloud computing further augment the capabilities of machine vision systems. The ability to connect to the internet and share data allows for real-time collaboration, remote monitoring, and the analysis of visual information. With the integration of cloud computing, machine vision systems can access vast computing resources and leverage sophisticated algorithms for complex tasks such as object recognition, scene understanding, and predictive analytics. This interconnected ecosystem facilitates seamless communication between cyber–physical systems, enabling humans to simultaneously interact with the virtual and real worlds [22].

## 2. An Overview of Published Articles

This Special Issue collates articles on information theory, measurement methods, data processing tools, and techniques for the design of machine vision systems and the instrumentation used in machine vision systems via the application of computer vision and image processing. Short summaries for each of the articles included within this Special Issue are provided below.

In the article by Garcia-Gonzalez et al. (contribution 1), a novel signal processing method is proposed for a technical vision system in order to deal with random fluctuations in electrical voltages during data acquisition, specifically the acquisition of an optoelectrical signal. An information theory-based method centering around the use of Shannon Entropy for extracting the features of optical patterns is presented to deal with the random processes presented in the acquisition of the signal. It is implemented in structural health monitoring to augment the accuracy of optoelectronic signal classifiers for a metrology subsystem of the technical vision system in order to enhance the system’s spatial coordinate measurement performance under real operation conditions in noisy electrical and optical environments, as well as to better estimate structural displacement and for an improved estimation of its health. In this study, five different machine learning (ML) techniques were used to classify the optical patterns captured. Linear predictive coding (LPC) and the autocorrelation function (ACC) were used for the extraction of optical patterns. The Shannon entropy segmentation (SH) method was used to extract relevant information from optical patterns, and the model’s performance was shown to be improved. The results reveal that segmentation with Shannon entropy achieved over 95.33% accuracy. Without Shannon entropy, the worst accuracy was 33.33%.

Wei et al. (contribution 2) propose a low-illumination image enhancement method based on structural and detail layer images to improve an image’s brightness while effectively maintaining the texture and details of the image, guaranteeing a high-quality image. A network called the SRetinex-Net model was designed and subsequently divided into two parts: a decomposition module and an enhancement module. The decomposition module mainly adopts the SU-Net structure, which is an unsupervised network that decomposes the input image into a structural layer image and detail layer image. The enhancement module mainly adopts the SDE-Net structure, which is divided into two branches: the SDE-S branch and the SDE-D branch. The SDE-S branch mainly enhances and adjusts the brightness of the structural layer image through Ehnet and Adnet to prevent insufficient or excessive enhancements of the brightness of the image. The SDE-D branch was denoised and enhanced with textural details through the use of a denoising module. The results of numerous experiments show that the proposed structure has a more significant impact on the brightness and detail preservation of restored images.

Stasenko et al. (contribution 3) present a promising approach for food quality control during the postharvest stage that leverages the power of Generative Adversarial Network (GAN) and Convolutional Neural Network (CNN) techniques to use synthesized and segmented Visible Near-infrared (VNIR) imaging data (“400–1100 nm”) collected under various environmental conditions (temperature and humidity) for early postharvest decay and fungal zone predictions, as well as for assessing the quality of stored food. Synthesized images were obtained via the pairing of Visible (V) “380–700 nm” images and Near-infrared (NIR) “780–2500 nm” images. By achieving accurate predictions and segmenting the decay and fungal zones, this approach offers significant advantages over traditional methods. NIR imagery provides detailed information about the diseased areas in stored fruits, which is why the hyperspectral cameras containing thousands of bands are used for food quality monitoring at postharvest stages. However, hyperspectral devices are expensive and are not suitable for use among farmers and sellers. Future research directions may include further comparisons with existing methodologies, exploring its applicability to different crops and storage conditions, and evaluating scalability for larger and more diverse datasets. The authors concluded that by harnessing deep learning (DL) and computer vision (CV) techniques in precision agriculture, significant strides forward in reducing food losses and ensuring a sustainable and secure food supply chain can be made.

Haipeng et al. (contribution 4) asserted that infrared and visible image fusion methods can be used to address the challenges of low-light scenes. This paper addresses the challenges of weak textural details, low-contrast infrared targets, and poor visual perception in existing deep learning fusion algorithms for low-light visible images to generate high-quality fused images under the conditions for such scenes. The authors propose a novel fusion method that exploits the characteristics of infrared and visible images to generate high-quality fused images under such conditions. The methodology followed consisted of the design of a Multi-Scale Edge Gradient Module (MEGB), which extracts texture information from both infrared and visible images. Additionally, they employed the Salient Dense Residual Module (SRDB) to extract salient features through pre-training with salient loss. The saliency map obtained from the SRDB was incorporated into the overall network training process. To fuse global and local information, the authors proposed the Spatial Bias Module (SBM). Extensive comparison experiments with existing methods were conducted to validate the effectiveness of the proposed approach in describing target features and global scenes. The results of the ablation experiments demonstrate the efficacy of the proposed modules. Furthermore, the authors evaluated the method’s facilitation for high-level vision tasks, specifically semantic segmentation in diverse low-light scene images. The proposed method was evaluated qualitatively and quantitatively on three datasets: TNO, MSRS, and M3FD. The authors compared their method with seven other fusion algorithms to demonstrate its superiority. The evaluation metrics used include Standard Deviation (SD), Visual Information Fidelity (VIF), Average Gradient (AG), Difference Correlation Sum (DCS), Entropy (EN), and Structure Fidelity (SF). However, the authors acknowledge that their method has limitations, including its inability to remove the overexposure effect caused by strong light interference. The results of the comprehensive evaluation and comparison experiments validate the proposed method’s superiority over existing algorithms.

Yichun et al. (contribution 5) aimed to reconstruct high-frequency details in the images of a scene by applying the thermal infrared image super-resolution method. They proposed an improved thermal infrared image super-resolution reconstruction method to solve the problem of poor image quality caused by the imaging mechanisms related to imaging sensors, such as motion blur, optical blur, and electronic noise, which lead to degradation in the quality of infrared images. The proposed method is based on multi-modal sensor fusion; as inputs, it uses low-resolution (LR) versions of infrared images, visible light images as the reference images, and high-resolution (HR) versions of infrared images to obtain a super-resolution (SR) image. Primary feature encoding, super-resolution reconstruction, and high-frequency detail fusion subnetworks were also included in this study. The network incorporates hierarchical dilated distillation modules and a cross-attention transformation module to extract and transmit image features effectively. A hybrid loss function was introduced to guide the network in extracting salient features from both thermal infrared and reference images while maintaining accurate thermal information. Additionally, a learning strategy is proposed to ensure high-quality super-resolution reconstruction performance, even in the absence of reference images.

The identification of text clusters under the sparsity of feature points derived from characters was achieved by Huei-Yung Lin and Chin-Yu Hsu in contribution 6. The proposed method was applied to invoices and banknotes for text region detection. The proposed approach involves the distillation of local image features combined with clustering analysis to identify meaningful regions of interest. This approach incorporates application-specific reference images for feature learning and extraction, enabling the identification of text clusters even in the presence of sparse character features. The method involves calculating clusters with high feature density and iteratively expanding the regions of interest for complete text coverage (feature extraction, clustering analysis, and region selection), enabling the detection of text clusters despite sparse feature points in real-world applications (adaptability to various application scenarios, including regions with different orientations, size changes, or perspective distortions), as it can achieve fast detection using limited computational resources. Unlike deep neural network approaches, it does not require extensive model training or high computational power, making it easily implementable with hardware-oriented acceleration. Additionally, a multi-stage algorithm with a robust receptor descriptor is presented for character recognition. The technique offers fast region detection and can be implemented with hardware acceleration. However, one limitation of the proposed approach is that its detection capability is limited to man-made structures. The authors state that their future work will center around investigating structural patterns in natural scenes, specifically for agriculture applications.

In contribution 7, Zheng, Siming, Mingyu Zhu, and Mingliang Chen propose a method called the hybrid multi-dimensional attention U-Net (HMDAU-Net) for reconstructing hyperspectral images from a single-shot 2D measurement in the context of spectral snapshot compressive imaging (SCI). The traditional methods for capturing spatial–spectral information involve scanning-based techniques, while SCI utilizes compressive sensing to capture 3D spatial–spectral data efficiently in a single measurement. However, the reconstruction process of retrieving the 3D cube from the 2D measurement is a challenging problem. The HMDAU-Net addresses this challenge by integrating 3D and 2D convolutions in an encoder–decoder structure, striking a balance between computational cost and performance. The network incorporates attention gates to highlight important features and suppress noise from skip connections. The authors observe that, for SCI reconstruction tasks, the depth of the backbone network (e.g., U-Net) is not as crucial as its width (number of kernels in each layer) in achieving good results. This observation is attributed to the difference in tasks between image reconstruction and image classification. Additionally, the attention gate is employed to extract essential correlations in the spectral data cube and improve the reconstruction performance of the network. Furthermore, the authors suggest that the HMDAU-Net could potentially be applied in tasks related to other domains, such as medical imaging, image compression, temporal compressive coherent diffraction imaging, and video compressive sensing.

As described by Pang, Xiyu, Yilong Yin, and Yanli Zheng in contribution 8, vehicle re-identification across multiple cameras is one of the main problems of intelligent transportation systems (ITSs) due to the small differences in appearance between vehicles of the same model and the significant changes in appearance that arise when viewing from different viewpoints. In this study, a model called multi-receptive field soft attention part learning (MRF-SAPL) was established by learning semantically diverse vehicle part-level features under different receptive fields through multiple local branches. In this model, soft attention is used to adaptively locate the positions of the vehicle parts on the final feature map, ensuring alignment and maintaining internal semantics. In particular, the soft-attention part learning module (SAPL) in this model does not require any part-related labels and can adaptively learn to localize the locations of the parts on the feature map to suppress severe spatial misalignments in vehicle Re-ID. A new loss function is proposed to obtain parts with different semantic patterns by penalizing overlapping regions. The main contributions of MRF-SAPL are flexible part-level feature learning, adaptive part localization using soft attention, and the use of multiple local branches with different receptive fields. The authors show that the model outperforms previous methods on vehicle re-identification datasets, demonstrating its effectiveness in learning fine-grained local features at multiple semantic levels to effectively distinguish different vehicles with similar appearances.

Junqing et al. (contribution 9) introduced an encryption scheme designed specifically for high-pixel-density images for ensuring the security of data transmission. The proposed scheme leverages the quantum random walk algorithm in combination with the long short-term memory (LSTM) model to address the efficiency- and statistical property-based challenges of generating large-scale pseudorandom matrices. The LSTM was divided into columns and utilized for training purposes. However, due to the random nature of the input matrix, effective training of the LSTM was not possible. To overcome this, the output matrix was predicted to possess a high level of randomness. This LSTM prediction matrix, matching the size of the key matrix, was generated based on the pixel density of the encrypted image, effectively facilitating image encryption. In terms of statistical performance, the proposed encryption scheme demonstrates an average information entropy of 7.9992, an average number of pixels changed rate (NPCR) of 99.6231%, an average uniform average change intensity (UACI) of 33.6029%, and an average correlation of 0.0032. Additionally, various noise simulation tests were conducted to evaluate the scheme’s robustness against common noise and attack interference in real-world applications. This approach harnesses the nearly infinite key space provided by the quantum random walk algorithm while addressing its low generation efficiency. Furthermore, the permutation and obfuscation processes in the proposed scheme make use of the key space of the quantum random walk, avoiding limitations related to the key space in a specific process.

Lei et al. (contribution 10) propose a novel method named NMYOLO for detecting infusion containers using the You Only Look Once version 4 (YOLOv4) approach to support medical staff in complex clinical environment by alleviating the pressure they face. The proposed method introduces several improvements to enhance the detection of infusion containers. First, a coordinate attention module was added after establishing YOLOv4 as the backbone to improve the model’s perception of direction and location of information. Next, the spatial pyramid pooling (SPP) module was replaced with the cross-stage partial spatial pyramid pooling (CSP-SPP) module, allowing for the reuse of input information features. Additionally, an adaptively spatial feature fusion (ASFF) module was added after the path aggregation network (PANet) to facilitate the fusion of feature maps at different scales. The method also utilizes the EIoU (Enhanced Intersection over Union) as a loss function to address the anchor frame aspect ratio problem, resulting in more stable and accurate detection. The experimental results reported in this article demonstrate the advantages of the proposed method in terms of recall, timeliness, and mean average precision (mAP). Although the proposed NMYOLO method achieved the desired detection performance, it has the drawback of reduced frame rate compared to YOLOv4. The authors suggest possible future improvements, such as using a lightweight backbone or removing the non-essential convolution modules to reduce the model’s parameters. They also mention the possibility of replacing modules or modifying the architecture to reduce the model’s size while maintaining its detection accuracy.

Shengping et al. (contribution 11) discuss the limitations of the Magnetic Flux Leakage (MFL) visualization technique used in the surface defect inspection of ferromagnetic materials when detecting complex defects, particularly cracks, and the loss of information during unidirectional magnetization. To address this problem, they propose a novel image registration method for MFL visualization that aligns images captured under different magnetization orientations. The method utilizes mutual information and Particle Swarm Optimization (PSO) to optimize the registration process. In this study, the design of a new registration method for MFL images under different magnetization orientations was achieved, a solenoid model was utilized in MFL image registration, and higher accuracy compared to traditional methods was demonstrated through comparative experiments, suggesting that the proposed method has the potential to enhance crack detection in MFL testing.

Jian et al. (contribution 12) introduce a one-stage scale enhancement pyramid network (SEPNet) to address the challenges of object detection in large-scale images captured by unmanned aerial vehicles (UAVs), particularly when detecting small objects with significant scale variation. The proposed SEPNet consists of two core modules: the context enhancement module (CEM) and the feature alignment module (FAM). The CEM module produces more salient context information by combining multi-scale atrous convolution and multi-branch grouped convolution to model global relationships and enhance object feature representation at different scales. It prevents the flow of features with lost spatial information into the feature pyramid network (FPN). The FAM module learns the transformation offsets of pixels to preserve aggregate feature space translation invariance, addressing feature inconsistency issues in the FPN. It also adaptively adjusts the location of sampling points in the convolutional kernel to preserve feature consistency and alleviate information conflict caused by the fusion of adjacent features. This module ensures that small objects are not drowned in feature conflicts. Additionally, this paper introduces channel attention to refine pre-aggregated features, allowing the network to focus on the target area rather than the background. Looking ahead, the authors of this paper suggest that designing lightweight structures for deployment on embedded devices could be a valuable topic to explore in future research. This implies a focus on optimizing the model’s efficiency without compromising its performance.

In conclusion, the application of information theory to computer vision and image processing represents a convergence of advanced technologies that bridge the gap between the virtual and real world. Through the integration of optoelectronic devices, sensors, artificial intelligence algorithms, embedded systems, robust control mechanisms, interconnectivity, big data, and cloud computing, machine vision empowers cyber–physical systems to collaborate with humans in their daily activities. As this field continues to evolve, we can anticipate a future where machine vision seamlessly integrates into our lives, unlocking new possibilities and transforming the way we perceive, interact with, and navigate both the physical and digital realms. The Guest Editors hope that after exploring the articles published in this Special Issue, entitled “**Application of Information Theory to Computer Vision and Image Processing**” (https://www.mdpi.com/journal/entropy/special_issues/MWI13854O7)—from the Information Theory, Probability and Statistics section of the ***Entropy* journal**—readers can take inspiration for their future research and publications.
